# Observations on Some Curious Physiological Phenomena Witnessed in a Case of Fever

**Published:** 1819-02

**Authors:** P. T. James


*For the London Medical and Physical Journal.*

### *Observations on some curious Physiological Phenomena witnessed in a Case of Fever;* by P. T. James, Esq.

A GENTLEMAN, aged 41 years, of particularly temperate habits, and of a very delicate and nervous constitution, was attacked, on October the 12th, (after travelling a journey of fifty miles in his carriage,) with slight rigors and a general feeling of lassitude. For these symptoms he took some aperient medicines. The next day the sensation of debility was increased: the skin was hot and dry, the tongue white and furred, and the bowels were much inclined to constipation. The heat of the skin was moderated by the plentiful application of cold water and air; the medicines consisted of aperients, salines, and sedatives. The febrile symptoms slowly increased. About the 18th, the abdomen became a little painful upon pressure with the hand, especially on the region of the liver. These symptoms were in part removed by leeches, blisters, and small doses of the blue-pill. The action of the bowels was very imperfect, and on the 21st they began to be distended with air. The bladder lost its power of acting; the use of the catheter became necessary night and morning. The head was not affected, the intellects being perfectly good. The pulse was about 110 in a minute, small, and rather hard. The skin was occasionally very hot, with great thirst. There was a tendency to paralysis; the lower lip being drawn a little to the left side, and the tongue, when thrust from the mouth, involuntarily pushed to the right side. The tumefaction of the bowels progressively increased upwards, with an increased difficulty of acting upon them with medicines.

I do not consider it necessary to detail the daily prescriptions, because medicines were productive of but little advantage.

The pulse, at this time (the 27th), became peculiarly variable; and, what I particularly wish to direct the reader's attention to, upon the introduction of ℥ ſs. of oil of turpentine into the rectum by enema, the pulse instantaneously fell in frequency from 120 in a minute down to 60, and, from a very small, it became a full and good pulse. In the space of a couple of hours, its frequency again increased, and again lost its strength. In the evening of this day, the artery beat, in the right wrist, about 110 in a minute, very small and feeble; at the same time the pulsation in the left wrist was full and strong, but of the same frequency. My attention was forcibly drawn to this peculiarity; and I was much surprised to find, in a few minutes, the strong and full pulsations were changed to the right arm, while the artery in the left became so small and feeble as scarcely to be felt: and it is remarkable that the strong pulse frequently changed from one arm to the other during the remainder of the patient's life, which was terminated on the 29th.

A peculiarity occurred in the temperature of the skin a few hours previous to death: the fingers of the left hand were quite warm, the hand itself quite cold, the fore-arm warm.

I feel the greater satisfaction in the relation of this case, because it came under the observation of Dr. Wilson Philip, who saw and prescribed for the patient. He observed to me, that he had never seen nor read of a case in which the influence of the nervous system on the blood-vessels, independently of any action of the former on the heart, was more strikingly displayed. It appears, from the experiments related in his Enquiry into the Laws of the Vital Functions, that the power of the blood-vessels may not only be influenced, but wholly destroyed, by affections of the nervous system. In another respect, this case appears to illustrate, in a striking manner, the results of his experiments. From them it appears, that the ganglion nerves are distributed as extensively as those proceeding directly from the brain and spinal marrow, the former influencing the blood-vessels through every part of the system; and that these two nervous systems may be separately affected. In the case before us, it was evidently the ganglion system that was affected. The slight affection of the muscles of voluntary motion, which appeared in the affection of the lip and tongue at an early period, quickly disappeared on the application of a few leeches to the temple; and the patient, to the last, retained the perfect use of all these muscles and of his intellects: but the bowels, the bladder, and the heart, (all of which derive their nerves from the ganglion system,) were greatly and permanently deranged,—the two former soon wholly and irrecoverably losing their power. The state of the temperature in the left arm previous to death was also very remarkable. It appears, from Dr. Philip's experiments, that the evolution of heat in the animal body is under the influence of the ganglion system. Mr. Brodie had formerly shown that it was influenced by the nervous system.

The case above related I consider not less striking than that quoted by Dr. Wilson Philip from Dr. Parry's work on the Pulse; where all voluntary power was lost in one arm, in which, however, the heat and pulse were natural; while the other arm was cold and without any pulse, but possessing complete voluntary power.

*Hereford; December* 3, 1818.

